# Investigating the Role of Verbal and Non-Verbal Working Memory Measures in Complex Sentence Comprehension by School-Aged Greek Speaking Children

**DOI:** 10.5964/ejop.18071

**Published:** 2026-08-28

**Authors:** Gavriil Karavasilis

**Affiliations:** 1 Department of Educational Sciences and Social Work, University of Patras, Patras, Greece; Stanford University, Stanford, USA

**Keywords:** verbal working memory, non-verbal working memory, sentence comprehension, complex sentences, children

## Abstract

**Objectives:**

The present study examines the role of Working Memory (WM), as a cognitive system of limited storage—processing resources, in complex sentence comprehension by typically developing children in both the verbal and non-verbal domain. In particular, the study compares non-verbal and verbal measures of WM in regard to their contribution to complex sentence comprehension.

**Methods:**

Participants were 169 children aged 8 to 12 years, native speakers of Greek. A self-paced reading task was used to assess the comprehension of syntactically complex sentences. The sentences tested were object-relatives. Non-verbal short-term memory (nvSTM) and non-verbal working memory (nvWM) was measured by corsi blocks forward and corsi blocks backward, respectively. Forward digit span was used for the measurement of verbal short-term memory (vSTM) and backward digit span for the assessment of verbal working memory (vWM).

**Results:**

Results showed that the only significant predictor of children’s comprehension was backward digit span.

**Conclusions:**

Findings suggest an important role of vWM in the comprehension of syntactically complex structures, lending support to the view that language comprehension is underpinned by a domain-specific cognitive system specialized to verbal processing.

## Defining the Working Memory Cognitive System

Working memory (WM) is the cognitive system for the short-term storage and processing of the information that enters human perception from environment in order individuals to perform a task (Baddeley, 2012; Just & Carpenter, 1992; Kane et al., 2007). According to Baddeley’s multicomponent view (Baddeley, 2012), WM is segmented into four distinct subsystems that co-operate with each other. Two of them are responsible for the short-term storage of information. The Phonological Loop (LP) is in charge of temporarily storing verbal items, whereas the Visuo-Spatial Sketchpad (VS) is responsible for the temporal storage of non-verbal stimuli. Both represent the concept of short-term memory (STM), the LP as verbal short-term memory (vSTM) and the VS as non-verbal short-term memory (nvSTM). In general, the term STM is used to describe the temporal storage of information, namely keeping information in an active state in memory ready for further processing (Baddeley, 2012; Cowan, 2008). A third component of Baddeley’s model is the Central Executive (CE). It oversees both the temporal storage and the processing of information. In this subsystem, information is being processed under execution operations held by the attentional/ executive control such as focusing attention, shifting attention, inhibiting irrelevant information while prioritizing relevant information, and applying specific processing strategies to the ongoing task (Baddeley, 2012). The CE could be viewed as the “working” part of the working memory cognitive system. Thus, the co-operation between the LP and the CE could be seen as verbal working memory (vWM), i.e., temporal storage of verbal items (LP) while processing them (CE); and the synergy between the VS and the CE as non-verbal working memory (nvWM), i.e., short-term storage of non-verbal information (VS) while processing it (CE). The fourth subsystem in the multicomponent model is considered as an interface in which input information, that is temporarily stored in STM, and relevant knowledge, that is permanently stored in LTM, are merged to facilitate processing (Baddeley, 2012; Cowan, 2019). This subsystem is named after the “Episodic Buffer” (EB) (Baddeley, 2000). In the present study I will use the terms vSTM (LP), nvSTM (VS), vWM (LP+CE), and nvWM (VS+CE) to refer to the different aspects of WM as described above.

## Working Memory Capacity and Complex Sentence Comprehension

Working memory is a cognitive system of limited resources. The WM subsystems are capacity—limited in terms of i) items, as only a small number of unconnected items can be simultaneously held in memory active (i.e., stored in STM) for processing (3–5 items, Cowan, 2001), and ii) time, as items are kept active in memory (i.e., stored in STM) for a very short period of time until decay occurs (Cowan, 2001, 2005). Another capacity restriction of the cognitive system (WM) has to do with the functioning of the attentional/ executive control. The attentional/ executive control is considered to have its own capacity limitations in terms of storage and in terms of how many operations it can efficiently perform in a certain period of time (Cowan, 2005, 2019).

According to the capacity-limit approach of language comprehension (Just & Carpenter, 1992; Waters & Caplan, 1996), children with lower WM capacity are expected to underperform in complex sentence comprehension measures in comparison to children with higher WM capacity. In complex sentence comprehension research, children are tested in thematic roles assignment. They are asked to decide which sentence referent has the thematic role of the agent of the action (/the Subject) in a manner of “who does what to whom”. Object-relatives (ORs) is the most common type of syntactically complex sentences that is used. Usually, ORs are presented along with subject-relatives (SRs) as syntactically simpler counterparts (e.g., Arosio et al., 2011; Arosio et al., 2012; Booth et al., 2000; Boyle et al., 2013; MacDonald et al., 2020) or as fillers (e.g., Finney et al., 2014; Rusli & Montgomery, 2017). ORs are considered more complex than SRs in terms of the WM resources that are consumed for their comprehension.

According to the *Dependency Locality Theory (DLT)* (Gibson, 1998, 2000) the memory cost for connecting two elements, i.e., a moved NP and its trace, in a syntactic structure is determined by the distance between the two. For example, in ORs like *“The boy [that the girl sees _ ] chases the policeman.”* the syntactic relationship between the moved NP (the boy) and the RC verb (sees) is validated at a latter point, at the trace position (_), after the second NP (the girl) and the RC verb (sees) (*“The boy [that the girl sees (the boy)] chases the policeman.”*). That’s not the case for SRs (e.g., *The boy [that _ sees the girl] chases the policeman*), in which the syntactic relationship between the first NP (the boy) and the RC verb (sees) is validated at a closer point before the second NP (the girl) and the RC verb (sees) (e.g., *The boy [that (the boy) sees the girl] chases the policeman*). Thus, for ORs the NP trace needs to be kept in memory for a prolonged time until both NPs and the RC verb are processed, unlike for SRs.

## The Domain Specificity of the Cognitive System That Supports Language Comprehension

In psycholinguistic research, there are different views about the domain specificity of the cognitive system that supports language comprehension.

According to the capacity-limit approach (Just & Carpenter, 1992; Waters & Caplan, 1996) language comprehension is supported by a domain-specific cognitive system, one that is dedicated—specialized to the processing of verbal information, i.e., verbal working memory (Caplan & Waters, 1999; Just & Carpenter, 1992; Waters & Caplan, 1997, 2004). In this approach, two views have emerged. Just and Carpenter (1992) consider that there is a common pool of storage and processing resources that are simultaneously shared between all representational levels of verbal processing (phonology, syntax, semantics, discourse). In contrast, Waters and Caplan (1996) segment the domain-specific cognitive system even further suggesting that there are different verbal WM subsystems that support different representational levels of verbal information (syntax-first-interpretative processes, then all other representational levels—post-interpretative processes) and different aspects of verbal processing (offline, online). Close to this view, Martin et al. (2021) suggested different WM subsystems within the verbal domain that serve as separate storages for phonological, semantic and orthographic information. First phonological representations are activated by the perceived stimuli (e.g., spoken words) and then they spread activation to lexical—semantic and orthographic levels. Information can be held simultaneously in different buffers. Despite their differences, these views anticipate that verbal WM tasks will be significantly correlated with measures of complex sentence comprehension, unlike non-verbal WM tasks.

According to another view about the role of WM capacity in verbally-mediated tasks, language comprehension is underpinned by a domain-general cognitive system, i.e., a system of attentional—executive processes that is reinforced for all kinds of tasks in both the verbal and the visual domain (Conway et al., 2003; Conway & Engle, 1996; Engle et al., 1999; Kane et al., 2004; Unsworth et al., 2014). Based on this view, WM memory tasks with minimum verbal materials that assess dimensions of attention and executive functioning (e.g., attention switching, WM updating, inhibition, planning) should strongly correlate with complex sentence comprehension measures. More than that, this relationship between WM capacity and complex sentence comprehension should apply similarly for both verbal and visual WM measures, as the same unitary cognitive system is reinforced for both.

So far two studies (MacDonald et al., 2020; Rusli & Montgomery, 2017) have compared between verbal and non-verbal WM measures in regard with their impact on complex sentence comprehension. Rusli and Montgomery (2017) investigated whether “word and digit recall” as a verbal WM task would predict OR comprehension to a greater extent than a non-verbal WM task of similar processing operations. Participants were children aged 9-12 years. In “word and digit recall” (from the Auditory Working Memory subtest [WJ-III; Woodcock, McGrew, & Mather, 2001]): children listened to a series of mixed numbers and words and had to recall first the words in their serial order and then the numbers in their serial order. Non-verbal WM was evaluated by a spatial span task, of similar operations: children were presented with a series of non-sense shapes one after the other either on the top or the bottom of the screen; right after they had to recall in a serial order first the shapes at the top and then the shapes at the bottom of the screen. Results indicated that only the verbal WM task was a significant predictor of OR comprehension accuracy (picture-pointing task). Similarly, MacDonald et al. (2020) examined the roles of verbal WM and non-verbal WM in the comprehension of (object- / subject-) wh-questions (e.g., Where is the deer that the cow is chasing? / Where is the deer that is chasing the cow?). Children aged 4;5 to 7 years were tested. Verbal WM was counted as forward and backward digit span composite score. Non-verbal WM was measured by a visual n-back task in which children had to decide whether a location on the computer screen were previously presented a step back or not. Results showed that neither verbal WM (forward + backward digit span) nor non-verbal WM (visual n-back) was significantly correlated with comprehension accuracy (picture-pointing task).

## Method

### The Present Study: Aims and Research Questions

The aim of the present study is to compare between verbal and non-verbal measures of WM in regard to their impact on complex sentence comprehension in typically developing children. Based on the domain-specific view of the cognitive system that is involved in language processing, it is expected that verbal WM measures will have a stronger influence on complex sentence comprehension than non-verbal WM measures. Alternatively, based on the domain-general view regarding the cognitive mechanism that supports language processing, it is anticipated that non-verbal WM measures will have a similar impact on complex sentence comprehension with verbal WM measures. The research question is formed as follows: do measures of verbal STM/ WM predict complex sentence comprehension (OR accuracy) to a greater extent than measures of non-verbal STM/ WM, in typically developing (TD) Greek speaking children aged 8–12 years?

### Research Tools and Materials

#### Cognitive Measures

##### Forward Digit Span

The *“digit span forwards”* subtest of the Greek adaptation of the “*Wechsler Intelligence Scale for Children - 5th Edition”* (WISC-V GR) (Wechsler, 2017) was used as a measure of vSTM. Children were asked to recall a series of digits per try in the same serial order that it was presented to them. In each subsequent level of difficulty, the total number of digits in each series is raised by one item. In total, the task contains nine difficulty levels starting from series of two digits and ending at series of ten digits. Each level includes two series-recall attempts. The task was stopped after two consecutive false recalls inside the same difficulty level. Each correctly recalled series was rated with 1. The highest possible score of the task is 18.

##### Backward Digit Span

The *“digit span backwards”* subtest of the Greek adaptation of the “*Wechsler Intelligence Scale for Children - 5th Edition”* (WISC-V GR) (Wechsler, 2017) was used as a measure of vWM. Similar to *“digit span forwards”* children were asked to recall a series of digits per try, but this time in the reverse order from that it was presented to them. For each subsequent difficulty level, the number of digits to be recalled is raised by one item. The lowest level includes series of two digits and the highest level includes series of ten digits per try. Each level entails two series of items. A stop rule at two consecutive incorrect recalls inside the same difficulty level was applied. Each series that was correctly recalled was rated with 1. The highest possible score is 18.

##### Corsi Blocks Forward

The “Corsi blocks” task (Corsi, 1973), forward version, was used to measure nvSTM. More specifically, an implementation of this task in the software package “Psychology Experiment Building Language (PEBL): Psychological Test Battery - Version 2.1” (Kessels et al., 2000; Mueller & Piper, 2014) was utilized. Children were asked to recall a series of squares presented to them on a laptop screen by clicking the cursor on each of them in the same serial order. The task started with series of two squares as a first level of difficulty. At each subsequent level an additional square was added. The last and the most difficult level included nine squares. Each level included two trials. The task was terminated in the case that children made two consecutive errors in the same difficulty level. Correct answers were rated with 1. The highest possible score was 18 (2 trials x 9 levels).

##### Corsi Blocks Backward

The “Corsi blocks” task (Corsi, 1973), backward version (PEBL; Kessels et al., 2000; Mueller & Piper, 2014), was used as a measure of nvWM. Children were asked to recall a series of squares per try in the reverse order from that they were presented to them by clicking the cursor on each square. The task was structured in nine levels of difficulty. The first level started with two squares. In each subsequent level one more square was added in the series. The last level included nine squares. Each difficulty level contained two trials. The task automatically stopped when participants made two mistakes inside the same difficulty level. Each series of squares that was recalled correctly was scored with 1. The highest possible score was 18 (2 trials x 9 levels).

#### Sentence Comprehension Measure: Procedure and Materials

Children’s comprehension of syntactically complex sentences was assessed by a self-paced reading task (similar to Booth et al., 2000). Children were asked to read the sentences at their own pace and, after each sentence, to answer a comprehension question. Each time they pressed the “space” button on the laptop keyboard a new phrase of the ongoing sentence was revealed to them. A non-cumulative presentation was chosen: each next phrase that was revealed after pressing the “space” button substituted the prior phrase of the sentence. This way children could not look back and re-read specific points of the sentence, but rather they had to keep the syntactic phrases stored in STM—WM similar to processing oral sentences. Each sentence consisted of six segments – syntactic phrases. After each sentence had been read, participants were asked to answer a comprehension question regarding the agent of action (“who does what to whom?”). Children chose the agent among the two referents of the sentence by pressing between two buttons symmetrically located on the laptop keyboard (“a”/ “l”).

Experimental sentences in the self-paced reading task were twenty-five (25) object-relatives (ORs) that were centrally embedded into main clauses (MCs), as shown in (1). An equal number of subject-relatives (SRs) was used as syntactically simpler counterparts (see (2)). In each item the only difference between the OR and the SR was the case of the RC NP. For ORs the RC NP was in nominative case indicating its role as the agent of the action, whereas for SRs the RC NP was in accusative form hinting its role as the patient of the action. Otherwise, in each sentence-pair the two sentences were identical to each other. All sentences consisted of twelve (12) words. After each sentence had been read, a comprehension question was presented to participants, as shown in (3). All experimental sentences are presented in detail in the Supporting Information file of the publication.

1

**Table:** 

OR:	O kirios^NOM^	[pu	voithise	**o^NOM^ pateras^NOM^** mu]^RC^	kratuse	ena kuti^ACC^	me erγalia.
	The mister^NOM^	[who	helped	**my father^NOM^** ]^RC^	was holding	a box^ACC^	with tools.

2

**Table:** 

SR:	O kirios^NOM^	[pu	voithise	**ton^ACC^ patera^ACC^** mu]^RC^	kratuse	ena kuti^ACC^	me erγalia.
	The mister^NOM^	[who	helped	**my father^ACC^** ]^RC^	was holding	a box^ACC^	with tools.

3

**Table:** 

question:	Pios^NOM-MASC^	voithise?	A. O pateras mu/ B. O kirios
	Who^NOM-MASC^	helped?	A. My father/ B. The mister

### Participants

Participants were one-hundred sixty-nine (169) typically developing (TD) children aged between 7;83 and 12;33 years (mean age: 9;58 years). Seventy-eight (78) of them were boys (46.15%) and 91 were girls (53.85%). All children participated in the study followed the typical development in terms of language ability, as assessed by a standardized test of morphosyntactic ability. In particular, the subscale “Morphology – Syntax” of the Reading Test – A (Padeliadu & Antoniou, 2007) were administered to all participants. The measure includes Tasks 5–8. In Tasks 5 and 6, children are asked to read one sentence per try and transform the word placed inside the parenthesis in the right form in order to complete the sentence. The difference between the two tasks is that in Task 5 participants have to transform verbs into the proper tense, person, number, or aspect (clitic morphology), while in Task 6 they have to first compose two words into one (lexical morphology) and then to transform it into the proper gender, number, and case (clitic morphology). These two tasks contain in total 15 items. In Tasks 7 and 8, children are asked to correct the order of a series of words per each try in order to make a sentence with correct meaning. Thus, these two tasks are more focused on syntactic ability (i.e., word order). The difference between them is that in Task 7 each set of stimuli is accompanied by a facilitating picture which serves as a hint for the meaning of the target-sentence, whereas in Task 8 there no pictures at all. These two tasks include in total 12 items. Each correct answer was rated with 1. So, the maximum score of the subscale is 27. For every task a stop rule was applied when participants made 3 consecutive mistakes. Children who scored at or below the 20th percentile (≤ 0.20) for their age on the subscale were excluded from the study as suspected of facing language difficulties (indicative of Developmental Language Disorder). All participants were also typical readers. Reading ability was measured by the “Decoding” and “Fluency” subscales of the Reading Test – A (Padeliadu & Antoniou, 2007). The “Decoding” subscale consists of Tasks 1–3. Task 1 and task 2 assess the reading of nonwords and words, respectively. Children were asked to read as accurately and as fast as they can first a list of 24 nonwords (Task 1) and then a list of 53 words (Task 2). Both tasks were stopped in the case of 5 consecutive items have been decoded unsuccessfully. Each successfully decoded item was rated with 1. In Task 3 participants were asked to indicate which are the true words in sets of items which included both words and nonwords. Each true word recognized as true word was scored with 1, whereas each true word not recognized as true word took 0. Each nonword chosen as true word was rated with 0. No stopping rule was applied. The maximum score of the task is 36. In total, the maximum score of the subscale “Decoding” is 113. The “Fluency” subscale consists of Task 4. Children were asked to read a text as accurately and as fast as they can in a time period of one minute. Each word that was read correctly until the stop point (1 min.) was rated with 1. The score was estimated by counting the total number of words that the participant tried to read minus the words that was inaccurately read. The highest possible score of the task is 279. For each of the two subscales (“Decoding”, “Fluency”) a total score was extracted. Children who scored at or below the 20th percentile (≤ 0.20) for their age were excluded from the studies as facing reading difficulties (indicative of Specific Learning Difficulties with impairment in reading / dyslexia). After these screening procedures an initial number of two-hundred eleven (211) possible participants was reduced to the final sample of 169 participants. All children handed back consent forms signed by their parents prior to their enrollment in the study.

### Statistical Analyses

Correlation analyses were carried out as a preliminary statistical analysis in order to test the contribution of each WM measure separately. The main statistical analyses, examining the effects of multiple WM measures simultaneously along with the impact of age, were performed under General Linear Modelling (GLM) (Agresti, 2015). The statistical methods used are described in detail in the Results section. The software used for the statistical analyses is RStudio (RStudio Team, 2020).

### Data Availability

The materials that were administered to participants, the data collected from their responses along with a data dictionary, and the analysis script for the statistical analyses (R code) are all accessible in the repository OSF (see Karavasilis, 2025).

## Results

### Correlation Analyses

Descriptive statistics including mean, median, variance (var), standard deviation (*SD*), range, skewness, and kurtosis are provided in Table 1, showing children’s performance in each WM measure. The Shapiro-Wilk test was performed for each measure to check whether scores are normally distributed. Data distribution of the variables was also checked through histograms (visual inspection). Results of the Shapiro-Wilk test showed that data were not normally distributed for all variables (see Table 2). Thus, in order to test the relationship between WM measures and OR accuracy, Spearman’s correlation analyses were performed. First, a bivariate correlation analysis demonstrated that age (in months) was significantly associated with OR accuracy (S = 603062, rho = 0.2503, *p* < .001). Since age was a significant factor shaping OR performance, partial correlations between WM measures and OR accuracy were carried out partialling out the effect of age (in months). As it is demonstrated in Table 3, only backward digit span was significantly correlated with OR accuracy after controlling for age (estimate: 0.1957, statistic: 2.5718, *p* = .01). This result suggests an important role of verbal working memory (vWM) in complex sentence comprehension. All other WM measures were non-significant in regard to their impact on OR accuracy.

**Table 1:** Descriptive Statistics for all Measures (n = 169)

Variable	Indicator						
	Mean	Median	Var	*SD*	Range	Skewness	Kurtosis
Age (in months)	114.95	115	139.2716	11.80	94–148	0.2617	-0.6354
Forward digit span	0.4194	0.4444	0.0055	0.0742	0.2222–0.6111	0.1837	0.2764
Backward digit span	0.3913	0.3888	0.0091	0.0954	0.1666–0.6666	0.2018	-0.0412
Corsi blocks forward	0.3901	0.3888	0.0091	0.0952	0.0555–0.5555	-0.4586	-0.0586
Corsi blocks backward	0.3857	0.3888	0.0078	0.0880	0.1111–0.5555	-0.3442	-0.1943

**Table 2:** Shapiro-Wilk Test

WM measure	Estimates		
	W	*p*	Decision
Age (in months)	0.9750	0.0038*	Reject H_0_
Forward digit span	0.9436	3.048e-06*	Reject H_0_
Backward digit span	0.9815	0.0241*	Reject H_0_
Corsi blocks forward	0.9513	1.404e-05*	Reject H_0_
Corsi blocks backward	0.9729	0.0022*	Reject H_0_

*Note*. a = 0.05; H_0_: data are normally distributed; if *p* < .05, reject H_0._

signif. codes: 0 ‘***’ 0.001 ‘**’ 0.01 ‘*’ 0.05 ‘.’ 0.1 ‘ ’ 1

**Table 3:** Partial Correlations Between WM Measures and OR Accuracy (Controlling for Age) (n = 169)

WM measure	Estimates		
	estimate	statistic	*p*
Forward digit span	0.1098	1.4234	.1564
Backward digit span	0.1957	2.5718	.0109*
Corsi blocks forward	-0.0215	-0.2778	.7815
Corsi blocks backward	0.0688	0.8888	.3753

signif. codes: 0 ‘***’ 0.001 ‘**’ 0.01 ‘*’ 0.05 ‘.’ 0.1 ‘ ’ 1

### Regression Analyses

In order to answer the research question of whether verbal STM/ WM measures predict complex sentence comprehension (OR accuracy) to a greater extent than measures of non-verbal STM/ WM measures, multivariate regression analysis (with interaction effects controlling for age) was performed.

Before running the main analysis, correlation coefficients (Spearman’s rho) between all WM measures were calculated in order to check whether some of the variables are highly correlated with each other. A high correlation between two variables is indicative that these two measures do not differ much, i.e., they probably represent the same construct. Moreover, including two highly correlated variables in the same multivariate model runs the risk of (multi)collinearity. Thus, the inclusion of two highly correlated variables will be prevented in the following analysis. In line with psychological research, Cohen's (1988) conventions to interpret effect size were used (0 ≤ *r* ≤ 0.29: small correlation, 0.30 ≤ *r* ≤ 0.49: moderate correlation, 0.50 ≤ *r* ≤ 1.00: strong correlation). Spearman’s rho > 0.5 was considered as indication of strong correlation between two variables. As presented in Table 4, a strong correlation coefficient was found between corsi blocks forward and corsi blocks backward (rho = 0.67). Hence, using these two variables in the same regression model was prevented. Since corsi blocks forward represent STM and corsi blocks backward represent WM, two different regression analyses were performed, i.e., one for each subsystem (STM, WM), testing verbal vs non-verbal measures in each subsystem.

**Table 4:** Correlation Coefficients Between WM Measures (n = 169)

WM measure	1	2	3	4
1. Forward digit span	—	0.1100	0.3273	0.3988***
2. Backward digit span		—	0.0934	0.0427
3. Corsi blocks forward			—	0.6748***
4. Corsi blocks backward				—

signif. codes: 0 ‘***’ 0.001 ‘**’ 0.01 ‘*’ 0.05 ‘.’ 0.1 ‘ ’ 1

In particular, two multivariate GLMs with OR accuracy as the outcome were built. In the first one (“Model STM”), age (in months), corsi blocks forward and forward digit span were the predictors (independent variables), comparing between non-verbal STM and verbal STM in regard to their influences on OR accuracy. In addition, the interactions between age and corsi blocks forward, as well as between age and forward digit span were also included in the model, in order to better test the influence of age. In the second model (“Model WM”), the predictor variables were age (in months), corsi blocks backward and backward digit span in order to compare between non-verbal WM and verbal WM respectively, in regard to their impact on OR accuracy. The interactions between age and each WM measure were also included in the model (see the Supporting Information file of the publication for the R code of the two models).

Table 5 summarizes the results of the “STM model”. The model explained a total 5.6% variance of the data (adj. *R*^2^ = 0.056) which was statistically significant (*p* = .012). First looking at the interactions, neither the interaction between age and corsi blocks forward nor the interaction between age and forward digit span had a significant effect on OR accuracy. Regarding main effects, none was found too. Age, corsi blocks forward and forward digit span were not strong predictors of OR accuracy. These results indicate that non-verbal STM and verbal STM do not have a strong impact on complex sentence comprehension.

**Figure 1:**
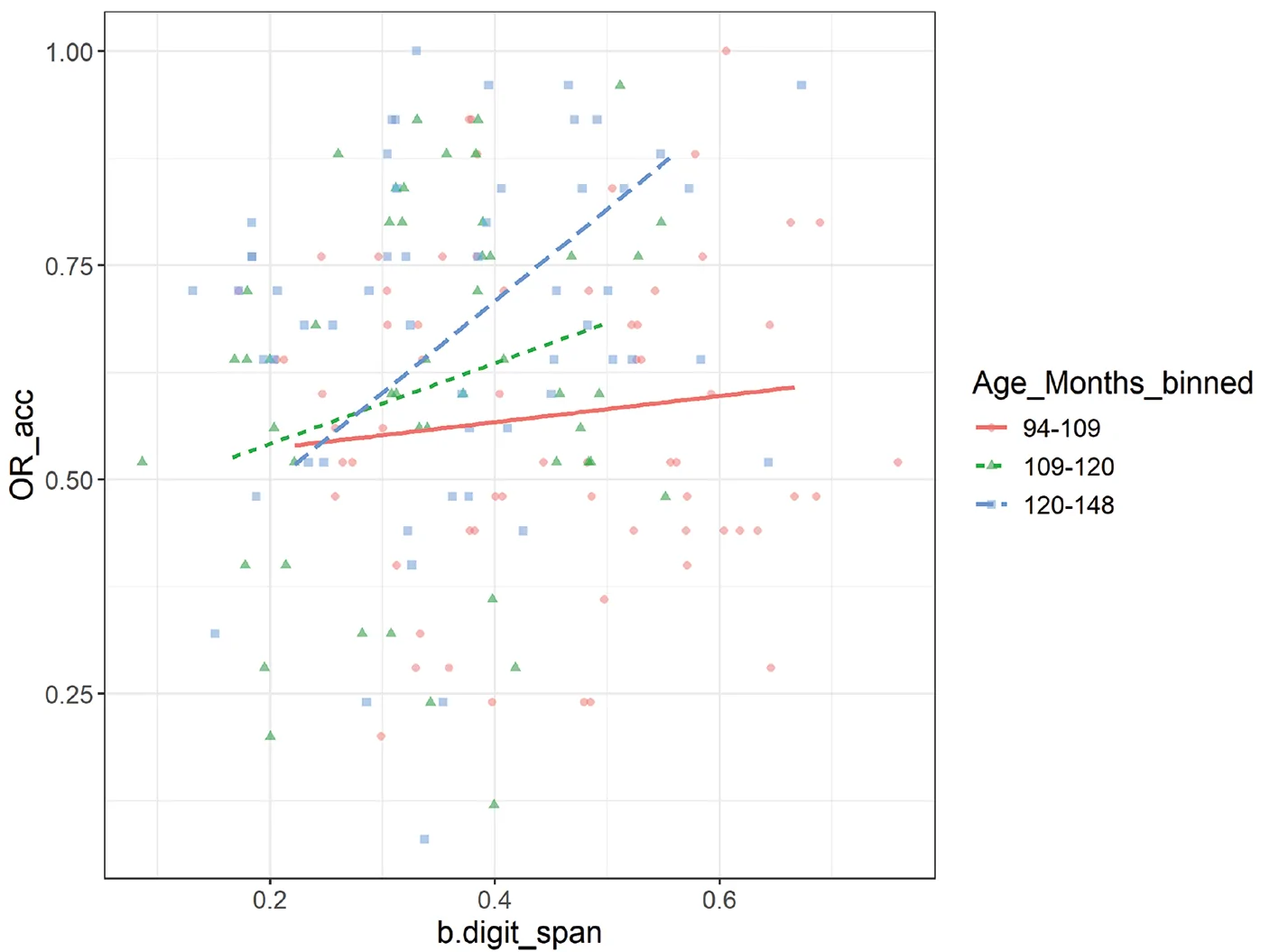
The Interaction of Age With Backward Digit Span on OR Accuracy

Table 6 sums up the results of the “WM model”. The model explained a total portion of 11.9% of the data variance (adj. *R*^2^ = 0.119) which was statistically significant (*p* < .001). An interaction effect was found. That was the interaction between age and backward digit span (*p* < .01). This finding suggests that as children are getting older and are developing higher level of vWM, they become better OR comprehenders. This is depicted in Figure 1, comparing between the youngest (94–109 months), the middle-aged (109–120 months), and the oldest (120–148 months) children of the study. That was the only significant interaction found. Regarding possible main effects, backward digit span was a strong predictor of OR accuracy over and above the other variables tested (*p* = .017). This finding suggests an important role of vWM in complex sentence comprehension. On the other hand, corsi blocks backward was not a significant predictor of OR accuracy. In sum, results indicate that vWM (along with age) has a strong relationship with OR accuracy, whereas non-verbal WM does not.

**Table 5:** Multivariate GLM With Interaction Effects for Verbal vs Non-Verbal STM (n = 169)

RESIDUALS	MIN -0.55200	1Q -0.11637	MEDIAN 0.02046	3Q 0.13888	MAX 0.41104
COEFFICIENTS	Estimate	Std. Error	*t* value	Pr(>|t|)	
(INTERCEPT)	0.684898	0.923934	0.741	0.46	
AGE (IN MONTHS)	-0.001336	0.008150	-0.164	0.87	
CORSI BLOCKS FORWARD	-0.596225	1.688973	-0.353	0.72	
FORWARD DIGIT SPAN	-0.684241	2.245926	-0.305	0.76	
AGE*CORSI BLOCKS FORWARD	0.003281	0.014686	0.223	0.82	
AGE*FORWARD DIGIT SPAN	0.009457	0.019264	0.491	0.62	
signif. codes: 0 ‘***’ 0.001 ‘**’ 0.01 ‘*’ 0.05 ‘.’ 0.1 ‘ ’ 1					
Residual Standard Error: 0.1954 on 163 degrees of freedom					
Multiple R-squared: 0.084, Adjusted R-squared: 0.056					
f-statistic: 3.018 on 5 and 163 *df*, *p*-value: .012					

**Table 6:** Multivariate GLM With Interaction Effects for Verbal vs Non-Verbal WM (n = 169)

RESIDUALS	MIN -0.55200	1Q -0.11637	MEDIAN 0.02046	3Q 0.13888	MAX 0.41104
COEFFICIENTS	Estimate	Std. Error	*t* value	Pr(>|t|)	
(INTERCEPT)	1.142935	0.887382	1.288	0.1996	
AGE (IN MONTHS)	-0.006414	0.007895	-0.812	0.4177	
CORSI BLOCKS BACKWARD	1.669153	1.668384	1.000	0.3186	
BACKWARD DIGIT SPAN	-4.247600	1.822121	-2.331	0.021*	
AGE*CORSI BLOCKS BACKWARD	-0.013786	0.014734	-0.936	0.3509	
AGE*BACKWARD DIGIT SPAN	0.041334	0.016085	2.57	0.0111*	
signif. codes: 0 ‘***’ 0.001 ‘**’ 0.01 ‘*’ 0.05 ‘.’ 0.1 ‘ ’ 1					
Residual Standard Error: 0.1889 on 163 degrees of freedom					
Multiple R-squared: 0.1362, Adjusted R-squared: 0.1097					
f-statistic: 5.139 on 5 and 163 *df*, *p*-value: .0002098					

### Conclusions

The goal of the present study was to examine verbal STM/ WM measures against non-verbal STM/ WM measures in regard to their impact on complex sentence comprehension in children aged 8 to 12 years. A series of correlation analyses, controlling for the effect of age, showed that backward digit span was the only significant correlate of OR comprehension accuracy. This finding demonstrates an important role of vWM (i.e., a subsystem of temporal storage and processing of verbal information) in the comprehension of syntactically complex sentences by middle-aged children (8–12 years old).

Furthermore, multivariate regression analyses were performed in order to identify which WM measure (among corsi blocks forward, forward digit span, corsi blocks backward, backward digit span) has the strongest effect on OR accuracy over and above the other WM measures. Two multivariate models were created, one for STM (i.e., temporal storage) and another one for WM (temporal storage + processing/ execution operations). In regard to STM (vSTM vs nvSTM), results of the multivariate analysis showed that none of the variables tested, in particular corsi blocks forward and forward digit span, was a significant predictor of OR performance. This finding suggests that complex sentence comprehension is not supported by a subsystem dedicated to temporal capacity, i.e., STM, both in the verbal and the non-verbal domain (vSTM, nvSTM). A non-significant role of STM was found to stand equally between vSTM and nvSTM. With respect to WM (vWM vs nvWM), multivariate analysis indicated that among the two predictors tested, in particular corsi blocks backward and backward digit span, only backward digit span was a significant predictor of OR accuracy. In addition, the interaction between age and backward digit was also significant suggesting an important mediating role of age. As children are getting older backward digit span is having a stronger effect on OR comprehension accuracy. These results are in line with that of the partial correlations showing than backward digit span was significantly correlated with OR accuracy after controlling for age. These findings suggest that complex sentence comprehension is underpinned by a subsystem of temporal capacity and processing/ execution operations (WM) that is specialized to the storage-manipulation of verbal information, i.e., vWM. Thus, the results of the present study lend support to the view that language processing is supported by a domain-specific cognitive system, i.e., a cognitive system that is dedicated – specialized to verbal processing (Caplan & Waters, 1999; Just & Carpenter, 1992; Martin et al., 2021; Shah & Miyake, 1996; Waters & Caplan, 1997, 2004). In contrast, the non-significant results for the non-verbal measure (corsi blocks backward) question the view that language processing is underpinned by a domain-general cognitive system, namely a central system of attentional-executive processes responsible for processing duties in both the verbal and the visual domains (Engle et al., 1999; Conway, Kane, & Engle, 2003; Conway & Engle, 1996; Kane et al., 2004; Unsworth, Fukuda, Awh, & Vogel, 2014). In line with the present study, MacDonald et al. (2020) found that nvWM (measured by a visual n-back task) was not significantly correlated with the comprehension of subject/object wh-questions in children aged 4;5 to 6;4 years. However, they also reported that vWM (measured by forward and backward digit span, counted as a composite score) was not a significant correlate of the sentence comprehension performance neither, showing a similar non-important role for both verbal and non-verbal measures, in contrast to the findings of the present study.

Until today, no other studies (apart from Rusli & Montgomery, 2017; MacDonald et al., 2020) have compared verbal and non-verbal WM measures in regard to their influence on the comprehension of syntactically complex sentences. In the present study we pursued to fill in these gaps. we found evidence that a domain-specific cognitive subsystem, that is verbal working memory (i.e., temporal storage and processing of verbal information), supports sentence-level comprehension in middle-aged children (8–12 years). In the present study we used backward digit span as a measure of vWM, a task that taps exclusively phonological processing (the phonological WM buffer; Martin et al., 2021). Future studies could compare verbal WM measures against visual WM measures in regard to their influence on complex sentence comprehension, using different tasks to segment further the verbal domain such as digit recall vs word recall. This way they would compare different verbal WM subsystems such as the phonological WM buffer vs the semantic WM buffer (Martin et al., 2021). The present study was limited in testing the verbal vs the non-verbal WM domain, as we did not address differences between Just and Carpenter's (1992) view of verbal working memory, Waters and Caplan’s (1996) view of verbal working memory, and Martin et al.’s (2021) view of working memory. This could be a path for future research.

## Supplementary Materials

The materials that were administered to participants, the data collected from their responses along with a data dictionary, and the analysis script for the statistical analyses (R code) are all accessible in the repository OSF (see Karavasilis, 2025).



KaravasilisG.
 (2025). Verbal vs non-verbal working memory measures that support complex sentence comprehension
[Data, code, materials]. PsychOpen. https://osf.io/wzrhy


## Data Availability

For this article, data is freely available (see Karavasilis, 2025).
